# Web-based self-management with and without coaching for type 2 diabetes patients in primary care: design of a randomized controlled trial

**DOI:** 10.1186/1472-6823-13-53

**Published:** 2013-11-16

**Authors:** Michael van Vugt, Maartje de Wit, Steven H Hendriks, Yvonne Roelofsen, Henk JG Bilo, Frank J Snoek

**Affiliations:** 1 Department of Medical Psychology, VU University Medical Center, Amsterdam, The Netherlands; 2 EMGO + Institute for Health and Care Research, VU University Medical Center, Amsterdam, The Netherlands; 3 Diabetes Centre, Isala Clinic Sophia, Zwolle, The Netherlands; 4 Department of internal medicine, University Medical Center Groningen, Groningen, The Netherlands

**Keywords:** Type 2 diabetes mellitus, Self-management, Web-based, Asynchronized coaching

## Abstract

**Background:**

Self-management is recognized as the cornerstone of overall diabetes management. Web-based self-management programs have the potential of supporting type 2 diabetes patients with managing their diabetes and reducing the workload for the care provider, where the addition of online coaching could improve patient motivation and reduce program attrition. This study aims to test the hypothesis that a web-based self-management program with coaching will prove more effective on improving patient self-management behavior and clinical outcome measures than a web-based self-management program without coaching.

**Methods:**

The effects of a web-based self-management program with and without coaching will be tested with a nested randomized controlled trial within a healthcare group in the Netherlands. In one year 220 type 2 diabetes patients will be randomized into an intervention group (n = 110) or a control group (n = 110). The control group will receive only the online self-management program. The intervention group will receive the online self-management program and additional online coaching. Participants will be followed for one year, with follow-up measurements at 6 and 12 months.

**Discussion:**

The intervention being tested is set to support type 2 diabetes patients with their diabetes self-management and is expected to have beneficial effects on self-care activities, well being and clinical outcomes. When proven effective this self-management support program could be offered to other health care groups and their type 2 diabetes patients in the Netherlands.

**Trial registration:**

Nederlands Trial Register NTR4064

## Background

Type 2 Diabetes Mellitus (T2DM) is a chronic metabolic disorder characterized by insulin resistance and beta-cell impairment [1]. Without proper treatment, T2DM can lead to long term complications such as neuropathy, nephropathy, retinopathy, cardiovascular disease, a poorer quality of life, and higher mortality rate [2]. The world prevalence of adults with diabetes in 2012 was estimated to be 371 million and is rapidly increasing [3]. In the Netherlands this number was estimated to be 1 million in 2012 and is increasing with 70.000 patients per year. The number of T2DM patients is expected to rise to over 1.300.000 in 2025, of which more than 90% will have T2DM [4,5]. The treatment of T2DM demands lifestyle changes and additional medication. When insulin is required patients need to self-monitor their blood glucose levels. Self-management is recognized as the cornerstone of overall diabetes management [6,7]. The Association of American Diabetes Educators (AADE) has defined 7 key self-management behaviors important for T2DM patients: healthy eating, being active, monitoring, taking medication, problem solving, reducing risks and healthy coping [8]. To promote this daily self-management for T2DM patients, educational and behavioral support programs have been developed and shown to be effective in terms of behavioral and medical outcomes [7,9-12]. Similarly web-based self-management programs have demonstrated improvements in health behaviors and health-related outcomes, and offer the possibility to increase both effectiveness and reach of clinical-based consultations [13-16]. Furthermore web-based self-management programs have the potential to decrease the workload of diabetes care providers. However it is unclear to what extent patients are motivated to use and adhere to online self-management programs. Face-to-face or telephone coaching can improve program adherence and online program effectiveness, both for medical and psychological outcomes. Moreover it can enhance satisfaction with the intervention [17-23]. To date, some studies that included coaching to their online T2DM self-management intervention showed improvements in dietary behavior, systolic blood pressure and a reduction in depressive symptoms [19,21,24-26] where as other studies found no effect [27,28]. However, the coaching in these studies targeted specific predefined health behaviors and instructions for diets and exercises. More flexible and adaptive style of coaching, attuned to the patients’ own chosen specific goals and health behaviors (and consequently possible multiple health behaviors), would seem more appropriate. To our knowledge this has not been investigated yet in T2DM patients. It is therefore unknown what the effect is of online (asynchronized) individual coaching on multiple goals and health behaviors as chosen by the patient. To our knowledge we would be the first to test the effectiveness of adaptive online asynchronized coaching in a web-based self-management program.

### Aims

We aim to test the hypothesis that self-management behaviors and biomedical outcomes improve in the group receiving an online self-management program with adaptive coaching versus those who don’t receive additional adaptive coaching. We expect online coaching to have neutral or favorable effects on well-being, quality of life and satisfaction with care. We will test our hypotheses using an existing patient web-portal, which offers diabetes information and an overview of personal clinical outcome measures, supplemented with a self-management support program (SSP) [29]. The combination of the web-portal with SSP is referred to as an interactive care platform (ICP) and will be discussed in further detail below.

## Methods

### Study design

**Figure 1:**
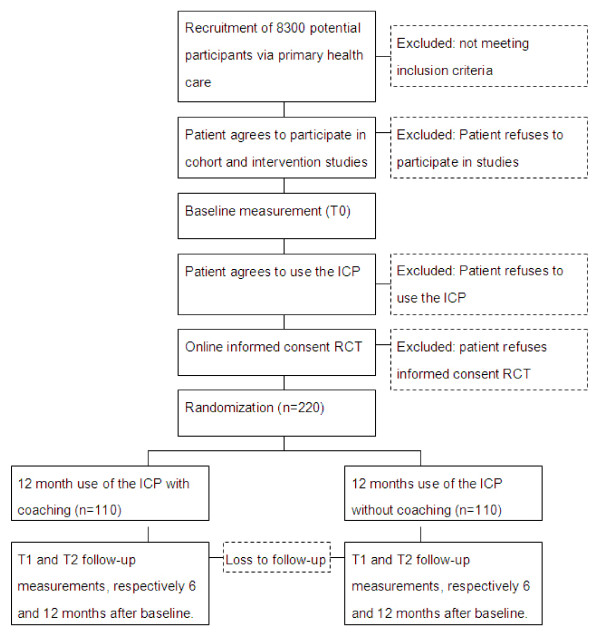
Flow chart of participants.

The current study design is nested within an ongoing cohort and intervention study, that focus on different constructs within the ICP [29]. We chose a two-arm nested randomized controlled trial (RCT) to test our hypotheses. Measurements are scheduled at three points in time: at baseline, 6 months and 12 months after baseline. During first log-on to the ICP, 220 patients (see power calculation) will be randomized into two groups. These groups are: 1) patients receiving the ICP with additional online coaching in the SSP, 2) patients offered only the ICP without coaching in the SSP. A visual representation of the situation is shown in Figure 1. The medical ethical committee of the VU University Medical Center (certified by the Central Committee on Research involving Human Subjects in the Netherlands) approved the study protocol.

### Recruitment

In line with the guidelines, the most patients in the care group are seen four times a year, of which one visit is the more extensive annual check-up. T2DM patients will be recruited when visiting their general practitioner (GP) or primary care nurse (PN) three months before the annual check-up. During their visit, PNs attend their patients to the existing studies, and to the availability of the ICP. After agreeing on participating in the studies and agreeing on using the ICP, patients receive a manual and login instructions for the ICP. During the first login to the ICP, patients are requested to provide additional informed consent.

### Study population

The sample consists of people with T2DM who are treated in primary health care. The available sample pool consists of approximately 8300 T2DM patients.

Inclusion criteria are: a diagnosis of T2DM, where the GP is defined as the main care giver; and aged ≥18 years. Exclusion criteria for the RCT are: Mental retardation or psychiatric treatment for schizophrenia, organic mental disorder or bipolar disorder currently or in the past. Insufficient knowledge of the Dutch language to understand the requirements of the study and/or the questions posed in the questionnaires. Life expectancy <1 year due to malignancies or other terminal illnesses. Cognitive impairment, including dementia, which interferes with trial participation. Any condition that the Investigator and/or coordinating Investigator feel would interfere with trial participation or evaluation of results.

### Description of the intervention

#### Development of Interactive care platform

For the creation of the ICP an existing patient web-portal was modified and merged with a SSP. This existing patient web-portal allows for a connection between health care practitioner and the patients, and allows patients to track their laboratory results as well as read personalized educational T2DM information online [29]. A SSP was developed and added to the patient web-portal to improve patients’ self-management, promote empowerment and patient autonomy, and to counter attrition of the web-portal [30,31]. The development of the SSP was informed by a literature review and previous experience in developing and testing theory-based self-management programs for people with diabetes [21,32,33]. Additionally the development of the SSP was guided by the Health Action Process Approach (HAPA) model of behavior change. This model integrates key features of social-cognitive theories of behavior change, and has strong empirical evidence from studies in the prevention and management of chronic diseases, including diabetes [34]. The HAPA model identifies self-efficacy, outcome expectancy, and risk-awareness, as key determinants of intention formation (motivation), while goal setting and planning are crucial for bridging the intention-behavior gap, such as actual practice of desired health behaviors, which allows for the formation of behavioral maintenance. Self-efficacy is an important determinant throughout the different stages of behavior change, including maintenance, relapse prevention and relapse management. Table 1 provides an overview of the application of the HAPA model and behavioral change techniques as proposed by Michie et al. (2011), as guidance for developing the SSP [35].

**Table 1:** Application of the HAPA model and behavioral change techniques used for the SSP development

**HAPA model component**	**Components used in the SSP**	**Used behavioral change techniques**
Risk awareness, outcome-expectancy, self-efficacy	- Information and education about T2DM in general and personalized to patients’ current health situation.	- Provide information on consequences of behavior in general/to the individual.
		- Model/Demonstrate the behavior.
	- Overview of patients’ personal clinical results.	- Prompt self-monitoring of behavioral outcome.
	- Patients’ clinical measures are compared to norm values and GPs’ advice, showed in a table and in a graph.	- Prompt review of outcome goals.
	- Information about the clinical measurements and information on behaviors that influence these clinical measurements.	- Provide information on consequences of behavior in general/to the individual.
		- Provide instruction on how to perform the behavior.
		- Model/Demonstrate the behavior.
	- Patients can fill-in a motivation why they want to change their behavior.	- Motivational interviewing.
Goal setting	- Patients are guided to choose a goal from a list of 4 behavioral goals (diet, exercise, medication & stop smoking)	- Goal setting (behavior).
Action planning, Self-efficacy	- Patients are guided to create a behavioral action-plan for the chosen goal. (Patients receive instructions and examples of action-planning in the SSP).	- Action planning.
		- Provide instruction on how to perform the behavior.
		- Facilitate social comparison.
		- Set graded tasks.
	**- Intervention group only**: Patients’ action-plan is send to a coach for feedback. The coach provides feedback (message in the SSP) on the process of action-planning, not on medical subjects. After receiving the feedback, patients are prompted to start the planned behavior.	- Provide feedback on performance.

	- Patients are prompted to start the planned behavior.	- Use of follow-up prompts.
Self-efficacy, Maintenance	- Patients receive reminders and encouragements via text-messaging and e-mail.	- Use of follow-up prompts.
		- Relapse prevention.
	- Patients receive a reminder to return to the ICP and evaluate their action plan.	- Set graded tasks.
		- Barrier identification/problem solving.
		- Use of follow-up prompts.
		- Provide feedback on performance.
	- Patients can fill in outcome measurements in the ICP	- Prompt self-monitoring of behavior/behavioral outcome.
		- Prompt review of behavioral goals/outcome goals.
	**- Intervention group only:** Patients can ask for feedback from a coach.	- Provide feedback on performance.

Content for the SSP was also partly derived from the successful PRISMA (PRoactive Interdisciplinary Self-Management) course, adapted by the VU University Medical Center for T2DM patients, which is based on the DESMOND-program developed in the United Kingdom [36-40]. In PRISMA patients are encouraged to set personal goals and formulate a realistic action plan that is then followed during routine clinical encounters with the diabetes care provider. Patients are prompted to monitor and evaluate their goals and behaviors, and make adjustments accordingly [36-39,41].

#### Self-management support program description

The ICP offers personalized information about one’s health status and educational modules that will inform the patient about risks related to specific behavioral and physical aspects derived from the collected personal clinical outcome data (HAPA: Risk-awareness). Furthermore within the SSP patients are stimulated to compare their personal clinical health outcomes to the GP’s advice and are triggered to think of ways to improve their health status. Additionally they receive information about relevant health related behaviors for improving these clinical outcomes (HAPA: Self-efficacy and Outcome expectancy). Based on this information, patients can choose behavioral goals derived from 4 of the 7 self-management behaviors defined by the AADE (diet, exercise, medication & stop smoking) where the other 3 defined self-management behaviors are integral part of the web-portal. In addition patients can compose self-chosen action plans to support these goals (HAPA: goal setting and planning). Patients receive automatic reminders and encouragements while they carry out their planned actions. Eventually patients are prompted to evaluate their behavioral goals and action plans, based on graded tasks and barrier identification with help from the SSP. After the evaluation, patients are encouraged to restart the behavioral goal setting and action planning (HAPA: maintenance loop).

### Intervention group

Hundred and ten randomized patients are offered the ICP with additional online (asynchronized) coaching to receive feedback on behavioral goals, action plans and evaluation of the executed health behaviors. These goals and planned health behaviors are chosen by the patient. The coaches follow a protocol where they serve as facilitator, and focus on process to specify action plans and let patients think for themselves. Coaches refrain from advising the patient with regard to medication or medical tests and refer to the GP if the patient requests such medical advice. After the patient has executed the planned health behaviors, the coach can support the patient by offering constructive and empowering feedback on the process of behavior change based on the HAPA model. Coaching will be carried out by trained master health-sciences students, by using asychronized messaging within the ICP. Feedback will be given on workdays within 48 hours after initial patient request.

### Control group

The remaining 110 randomized patients will receive the ICP without additional coaching.

### Outcome assessment

#### Primary outcome measures

Our ambition is to improve patients’ self-care activities, as measured with the Summary of Diabetes Self-Care Activities (SDSCA) questionnaire [41].

#### Secondary outcome measures

Diabetes distress will be assessed using the Problem Areas In Diabetes care (PAID) survey, 5-item version [42].

Emotional well-being: The five items (WHO-5) questionnaire covers positive mood, vitality and general interests and has clinical use as depression screening [43,44]. Health-related Quality of Life (HRQoL) is tested by using the EQ-5D questionnaire [45,46].

Health status: A selection of collected data during the regular yearly check-up will be included in the research to objectify a person’s health status. This includes the following parameters: glycemic control (HbA1c), Body Mass Index (BMI), systolic blood pressure, diastolic blood pressure, cholesterol and smoking status.

Medical care: For all participants in the study the actual medical care utilization and use of medication will be documented using the following parameters: number of encounters with care providers, number of hospitalizations, use of medication (based on prescribed medication) and patient profile data.

ICP use during the study period will be measured using: number of log-ons, time spent per session, number of educational modules taken, number of coaching feedback received, number of goals set and adjusted.

### Statistical analyses

Analyses will be conducted by using SPSS and Stata software. Normally distributed data will be presented as means and standard deviation, otherwise as median and interquartile range. Dichotomous/categorical data will be presented as numbers and percentage of total. After one year, the group of patients in the RCT who received additional coaching will be compared to the group of patients in the RCT who used the ICP without coaching on primary outcome, i.e. self care activities total score. To evaluate differences in target variables within and between groups over time we will use a linear mixed model for repeated measures (after 6 and 12 months). Baseline variables will be used as covariates. Analyses will be based on Intention-to-treat. Longitudinal linear regression, using Generalized Estimation Equations (GEE) will be used to investigate the differences between the two groups on primary (self-care activities) and secondary outcome variables over time. With GEE the relationships between the variables at different time-points are analyzed simultaneously, reflecting the relationship between the longitudinal development of the outcome variables and the longitudinal development of the predictor variables. GEE adjusts for the correlation between repeated observations taken in the same patient and has the advantage of handling longitudinal data on subjects with varying numbers of unequally spaced observations. The latter is important, because the assessments are scheduled within routine care and as a consequence, the time between the consultations can differ. Multi-level analyses will be applied to correct for the different primary care groups. All analyses will be corrected for baseline values, gender and age.

### Sample size calculation

Change in self-care behavior is the primary endpoint as measured by the SDSCA total score. The study of Thoolen et al. was used as reference [47]. Sample size calculations indicate that a sample of 131 patients is sufficient to demonstrate an effect size of 0.30 at a significance level of 5% with a power of 80%. Given an expected drop out rate of 20%, we will include at least 220 patients in our RCT, 110 patients in each group.

## Discussion

### Strengths and limitations

Integrating the ICP in routine primary care for T2DM patients, adds to the external validity of the study. Primary care physicians can use the intervention as support for the standard treatment of T2DM patients. Health care professionals play an active roll in recruiting, informing and motivate patients for using the ICP. By doing so, people who are unmotivated and who would initially not consider using an ICP could be recruited, which can further increase external validity. With the integration of the ICP in standard primary diabetes care, we hope to reduce attrition, which is a known problem for web-based interventions [48]. With the use of reminders and online contact between participants and coaches we hope to limit attrition rates.

The flow of patients in this study is limited by the three different informed consents and agreements patients have to sign before they can be randomized for the RCT. This can create a bias of including only highly motivated patients in the RCT study.

The study participants will consist of primarily older patients who may be lacking computer skills which could have affect participation and attrition rates of the intervention.

In the current study proposal, the measurements will be executed at baseline, 6 months and 12 months after baseline. Therefore long-term effects of the web-based intervention cannot be measured.

### Future implementation

The developed ICP is currently being tested in primary diabetes care setting. Having the support of a major Dutch insurance company increases the chances of the intervention being set out to other primary health care groups. Additional primary health care groups have already showed interest in using the ICP.

## Abbreviations

AADE: Association of American diabetes educators; BMI: body mass index; EQ-5D: EuroQol – 5 dimensions; GEE: Generalized estimation equations; GP: General practitioner; HAPA: Health action process approach; HRQoL: Health related quality of life; ICP: Interactive care platform; PAID-5: Problem areas in diabetes 5 questions; PN: Primary care nurse; RCT: Randomized controlled trial; SDSCA: Summary of diabetes self-care activities; SSP: Self-management support program; T2DM: Type 2 diabetes mellitus; WHO-5: WHO five item measure of well being.

## Competing interests

This study was funded by the foundation Health Within Reach (Zorg Binnen Bereik). The foundation has no role in writing or publication of this manuscript. They also have no role in study design, data collection, data analysis or reporting of study results. All authors declare that they have no competing interests.

## Authors’ contributions

MvV and MdW are researchers in this project and mainly drafted this manuscript, furthermore they developed the SSP that has been added to the ICP. YR and SH are other researchers in this project and helped to draft this manuscript. They were involved in the development and study of the original web-portal which was later transformed to the ICP. FJS and HJGB conceived the involved studies and participated in its design and coordination and helped to draft the manuscript. All authors read and approved the final manuscript.

## Pre-publication history

The pre-publication history for this paper can be accessed here:

http://www.biomedcentral.com/1472-6823/13/53/prepub
